# Hepatocellular carcinoma arising from hepatic adenoma in a young woman

**DOI:** 10.1002/ccr3.2975

**Published:** 2020-05-27

**Authors:** Haythem Yacoub, Hela Kchir, Dhouha Cherif, Hajer Hassine, Slim Haouet, Asma Ayari, Habiba Mizouni, Saber Mannai, Mohamed Tahar Khalfallah, Nadia Maamouri

**Affiliations:** ^1^ Gastroenterology B department La Rabta Hospital Tunis Tunis Tunisia; ^2^ Faculty of Medecine of Tunis El Manar University Tunis Tunisia; ^3^ Pathology Department La Rabta Hospital Tunis Tunis Tunisia; ^4^ Radiology Department La Rabta Hospital Tunis Tunis Tunisia; ^5^ Surgery Department Mahmoud El Matri Hospital Ariana Tunisia; ^6^ Surgery Department Mongi Slim Hospital Tunis Tunis Tunisia

**Keywords:** hepatectomy, hepatic adenoma, hepatocellular carcinoma, malignant transformation, oral contraceptives

## Abstract

Hepatocellular carcinoma (HCC) arising from hepatic adenoma is an infrequent situation. Only a few cases were reported in the literature. We present a rare case of hepatocellular carcinoma arising from HA in a young woman with no medication history of oral contraceptives. Surgical resection is the only available treatment.

## Graphical abstract

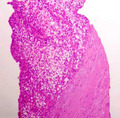

## 1 INTRODUCTION

Hepatocellular adenomas (HAs) are benign hepatic lesions that predominantly occur in young women using oral contraceptives. Most of these tumors are detected incidentally during imaging for not related pathology. However, patients may present with abdominal discomfort or pain, hepatomegaly, and intraperitoneal hemorrhage in case of spontaneous tumor rupture.^1^, ^2^ HAs can undergo malignant transformation to hepatocellular carcinoma (HCC) in rare cases (about 4%).^3^ Only a few cases regarding HCC arising in HA in female patients have been reported in the literature. For most cases of hepatocellular carcinoma arising in HA, there is a history of oral contraceptives use.^4^ Here, we report a rare case of hepatocellular carcinoma arising from hepatocellular adenoma in a young woman who had not taken oral contraceptives. This case was reported to keep in mind that hepatic adenoma carries a risk of malignant transformation.

## 2 CASE REPORT

**FIGURE 1:**
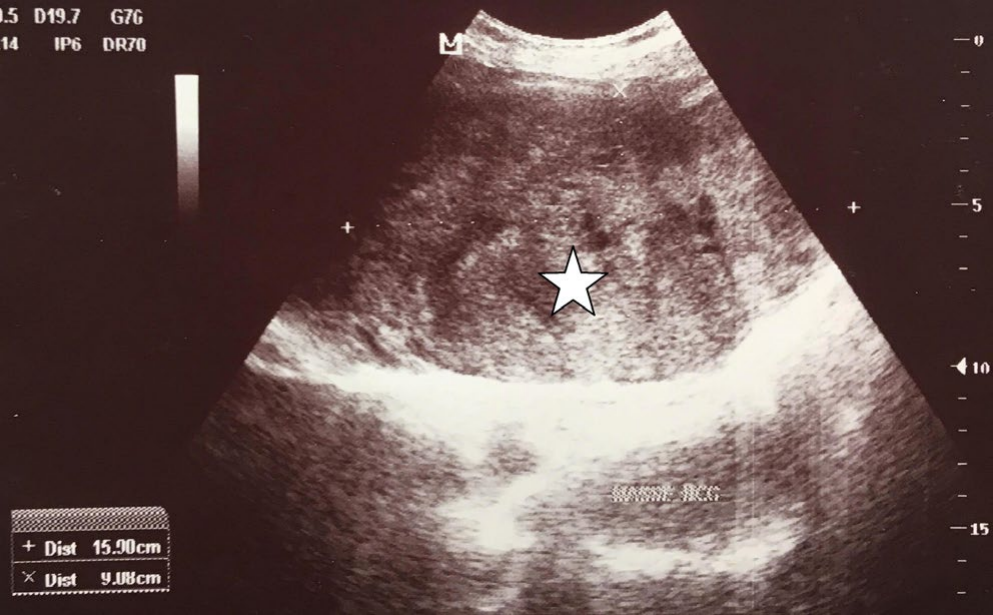
Epigastric mass (15 × 9 cm) on abdominal US (asterisk)

A 35‐year‐old married housewife, who lives with her husband and her three children in the city, was referred to us because of right upper quadrant abdominal discomfort and intermittent fever for the last 6 months. There was no history of drug or alcohol intake. Her background was significant for cesarean section, with no history of any medication or oral contraceptive use. On examination, an immobile and tender palpable epigastric mass was found, the temperature was 37.2°C, blood pressure was 120/80 mm Hg and pulse was 70 bpm. Abdominal ultrasound revealed a 15 × 9 cm sized epigastric mass (Figure 1).

She then underwent a gadoxetate‐enhanced magnetic resonance imaging revealing a 15 × 11 × 9 cm heterogeneous encapsulated lesion arising from the left hepatic lobe. This lesion was hypointense in T1‐weighted imaging and hyperintense in T2‐weighted imaging. On the arterial phase, a contrast enhancement was noted. Washout was observed on the portal and delayed phases. These findings were suspicious for HCC.

**FIGURE 2:**
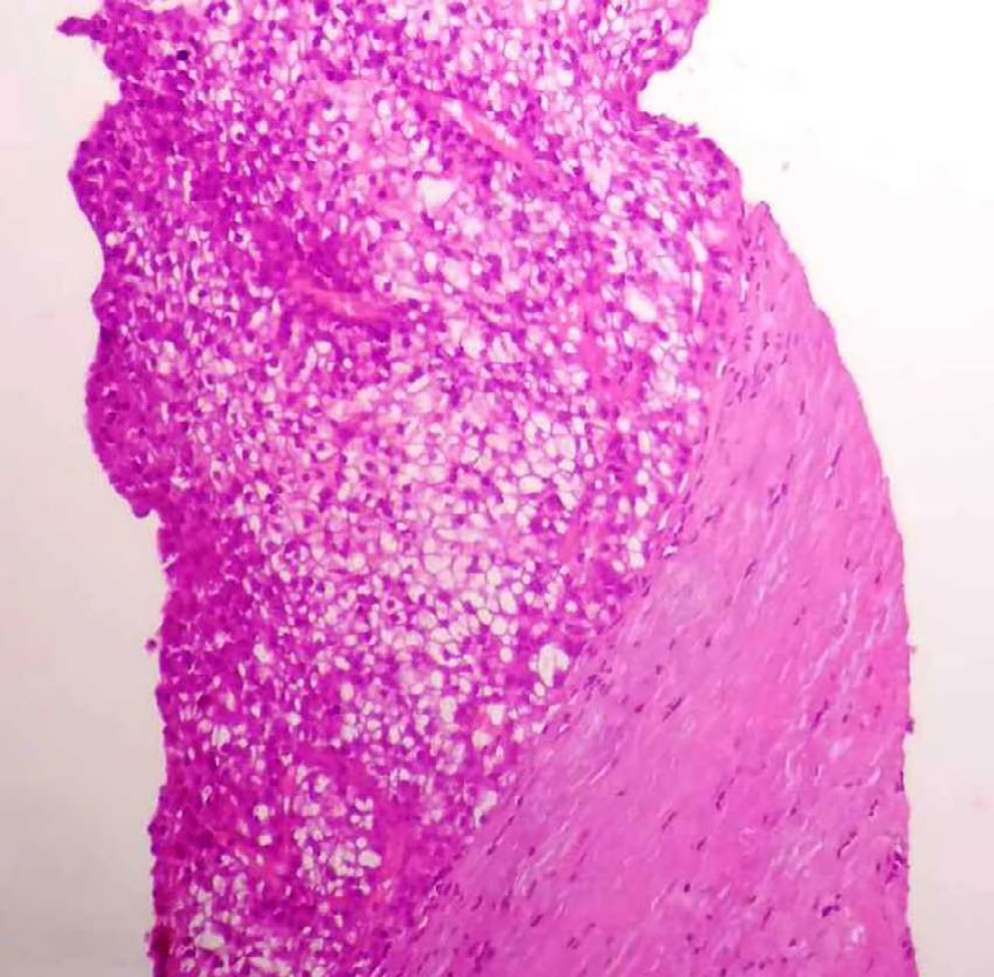
Trabecular pattern made of thin plates (1‐3 hepatocytes)

**FIGURE 3:**
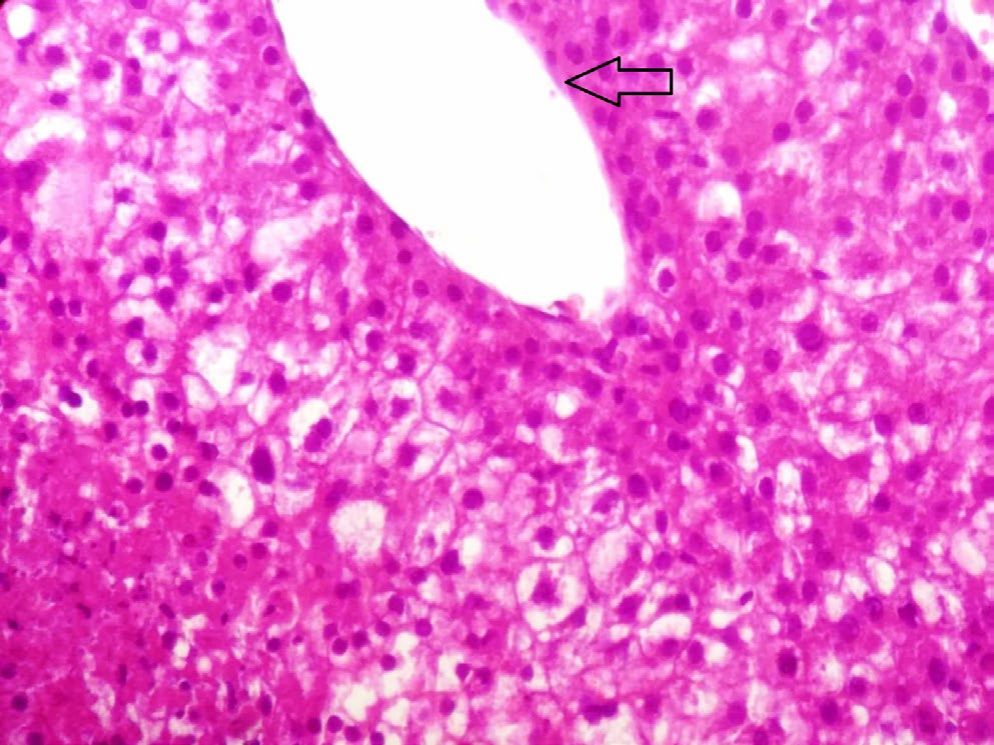
Sinusoidal vessel surrounding tumor cells (arrow)

**FIGURE 4:**
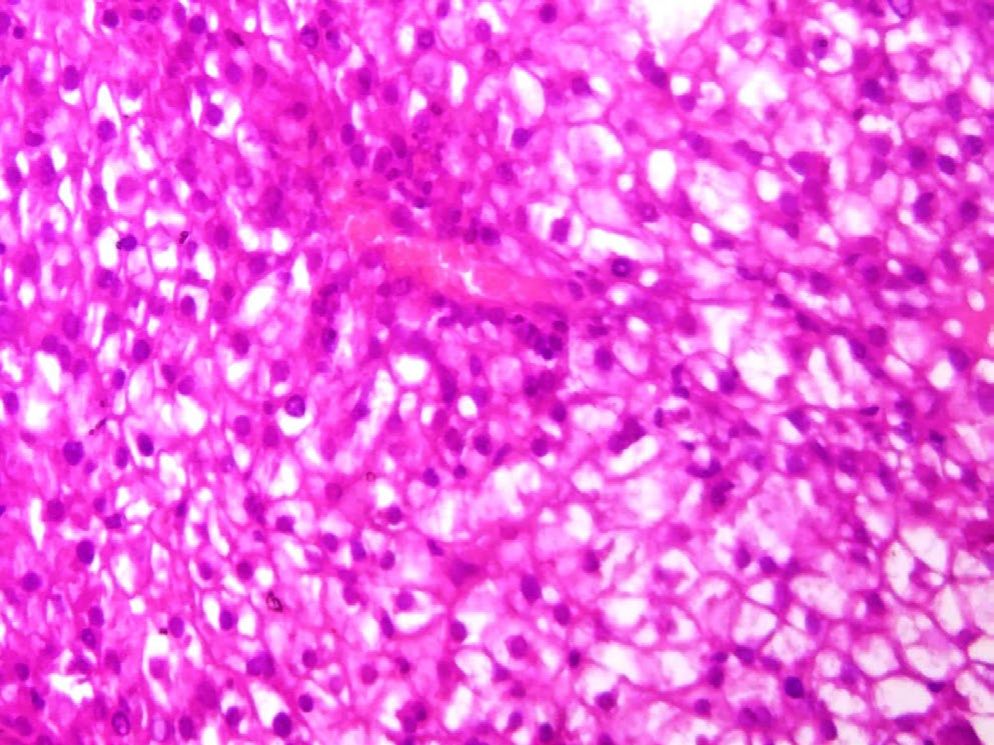
Polygonal cells with abundant granular eosinophilic cytoplasm, high nuclear‐cytoplasmic ratio, round nuclei with coarse chromatin

Liver function tests showed serum alkaline phosphatase (ALP) levels about two times the upper limit of normal (230 IU/L, N < 113 IU/L). Gamma‐glutamyl transferase (GGT), aspartate aminotransferase (AST), alanine aminotransferase (ALT), and total bilirubin levels were normal. Other parameters, including leukocytes, hemoglobin, platelet counts, and renal function tests, were normal. Serum tests for human immunodeficiency virus (HIV) and hepatitis C virus were negative. Hepatitis B markers were positive for hepatitis B surface antigen and hepatitis B core antibody. The viral load was undetectable. Alpha‐fetoprotein level was very high (19 960 IU/L, N < 5 IU/L). Tests for serum tumor markers were negative for CEA and CA19‐9. Fine needle aspiration cytology showed typical features of trabecular hepatocellular carcinoma including liver cell cords that were one to three cells thick and nuclear atypia (Figures 2, 3, 4) associated with adenoma features including liver cell cords that were one to two cells thick and increased cell density (Figure 2). Nontumor tissue showed chronic hepatitis A1F0 (A1—mild activity, F0—no fibrosis) according to METAVIR score. Based on the radiological and histological findings, the patient was diagnosed with HCC arising from hepatic adenoma and left hepatectomy was carried out. The postoperative course was uneventful. After 7 years of follow‐up, the patient is in good condition. The hepatitis B viral load was undetectable at each annual control, and no treatment was indicated.

## 3 DISCUSSION

We reported a 35‐year‐old woman with no medical history of oral contraceptives use, who presented with abdominal pain and a palpable abdominal mass. Gadoxetate‐enhanced MRI revealed a 15 × 11 × 9 cm heterogeneous encapsulated lesion arising from the left hepatic lobe. Pathological analysis of the tumor revealed HCC arising from hepatic adenoma. The patient underwent a left hepatectomy with no complications. Clinical and abdominal ultrasonography follow‐up revealed no evidence of recurrent disease. The originality of this case report stems also from the fact that hepatic adenoma malignant transformation occurred in a hepatitis B inactive carrier with no cirrhosis.

Hepatic adenomas are uncommon, rare benign liver neoplasms that occur more frequently in young women with a medication history of oral contraceptives. The incidence of HAs is 3‐4 per 100 000 women.^1^ A long duration of contraceptive use increases the risk of developing HA.^5^ This tumor can also occur in a patient with type 1 glycogen storage disease, iron overload, and men taking anabolic steroids.^6^, ^7^ Hepatitis B is not known as a risk factor for HA. Nevertheless, hepatic adenomas may also affect men without risk factors.^8^ However, it is interesting that our patient did not have any risk factors for HA such as metabolic disease, medication history of oral contraceptives, or steroid hormone. Hepatic adenomas usually arise in the right hepatic lobe. They are generally solitary with a tendency for hemorrhage.^9^ Occasionally, they may be multiple and reach up 30 cm in diameter.^8^, ^10^ The ultrasound of the liver usually shows a hepatic mass but features for hepatic adenoma are nonspecific on it. Multiphase CT scan is able to demonstrate characteristic findings for hepatic adenoma, but in some cases, MRI with liver‐specific contrast agent is required to lead to a final diagnosis.^11^ The histological findings are regular proliferation often in trabeculae of normal appearing hepatocytes with compressed sinusoids in between with absent bile ducts.^8^, ^12^

Although HAs are benign lesions, they do have a potential of malignant transformation. The adenoma‐carcinoma sequence is defined by the presence of foci of dysplasia within adenomas, which predispose to carcinoma in situ and epithelial cancers.^11^ A systematic review of the literature identified a total of 24 case reports concerning the malignant transformation of HAs.^10^, ^11^ The exact frequency of the malignant transformation is hard to be determined, but its frequency was 4.2% among all adenomas on a systematic review, including 1635 adenoma cases.^10^ The average age of patients with malignant transformation was 41 years (range, 19‐70). Fourteen of these patients (74%) were women, and five were men (26%). Mean time between the start of oral contraceptives use and HA diagnosis was 14 years. The mean size of transformed adenomas is 10.5 cm (4.5‐18 cm). Twelve women of the 19 reported to having malignant alteration presented with a history of contraceptive usage.^10^ Only a few women with no oral contraceptive medication presented malignant transformation.^10^, ^12^ Only 16% of patients among the 19 presented with multiple HAs. In most cases of HCC arising in HA, the malignant transformation was concomitant to the diagnosis of HA, as in our patient. A literature search showed that only three cases of malignant alteration happened in a tumor less than 5 cm in size.^10^ It is important to determine associated factors with malignant transformation in HAs. As already mentioned, HAs larger than 5 cm do have the potential of malignant transformation. Patients with type I glycogen storage disease, those with ongoing use of steroids, and male patients are at high risk of malignant transformation.^13^ Several studies reported that the ß‐catenin gene is implicated in malignant transformation (10%‐15%). The ß‐catenin activation deregulates the ß‐catenin pathway, which is known to play a determinant role in the proliferation of liver cells.^10^, ^13^ Hepatitis B‐induced HCC differ from HA transformation into HCC. The viral X gene has an important role as it is a very powerful transactivator for transcription of many oncogenes.^14^ Some researchers recommend HA resection only for adenomas larger than 5 cm in diameter based on the fact that HA lesser than 5 cm or that are not increasing in size may regress over time, especially after hormonal contraception withdrawal.^15^ However, as some cases of malignant transformation have been reported in HA measuring less than 5 cm, some others recommend surgical resection regardless of the HAs size.^10^, ^15^ Surgical treatment is also recommended in male patients regardless the size of HA, patients with rising alpha‐fetoprotein, and adenomas in glycogen storage diseases.^16^, ^17^ However, the malignant transformation may happen without an increase in alpha‐fetoprotein levels or tumor size.^17^

## 4 CONCLUSION

Hepatic adenomas are uncommon, benign liver lesions typically affecting young women of childbearing age. Despite the fact that they are benign neoplasms, they can become malignant. Rare cases of hepatocellular carcinoma arising from hepatic adenoma with no history of oral contraceptives use were reported in the literature. Surgical resection of hepatic adenoma is the only treatment available for this type of tumor.

## CONFLICT OF INTEREST

None declared.

## AUTHOR CONTRIBUTIONS

Nadia Maamouri: contributed to concepts; Haythem Yacoub, Nadia Maamouri, and Hela Kchir: contributed to design; Haythem Yacoub, Nadia Maamouri, Slim Haouet, and Hela Kchir: contributed to definition of intellectual content; Haythem Yacoub: contributed to literature search; Haythem Yacoub, Nadia Maamouri, and Asma Ayari: contributed to manuscript preparation; Nadia Maamouri and Haythem Yacoub: contributed to manuscript editing; Haythem Yacoub, Hajer Hassine, Nadia Maamouri, Hela Kchir, Slim Haouet, Habiba Mizouni, Mohamed Taher Khalfallah, and Sabeur Mannai: contributed to manuscript review; Haythem Yacoub served as guarantor.

## PATIENT'S CONSENT

Yes.
